# Computational neurorehabilitation: modeling plasticity and learning to predict recovery

**DOI:** 10.1186/s12984-016-0148-3

**Published:** 2016-04-30

**Authors:** David J. Reinkensmeyer, Etienne Burdet, Maura Casadio, John W. Krakauer, Gert Kwakkel, Catherine E. Lang, Stephan P. Swinnen, Nick S. Ward, Nicolas Schweighofer

**Affiliations:** Departments of Anatomy and Neurobiology, Mechanical and Aerospace Engineering, Biomedical Engineering, and Physical Medicine and Rehabilitation, University of California, Irvine, USA; Department of Bioengineering, Imperial College of Science, Technology and Medicine, London, UK; Department Informatics, Bioengineering, Robotics and Systems Engineering, University of Genoa, Genoa, Italy; Departments of Neurology and Neuroscience, John Hopkins University School of Medicine, Baltimore, MD USA; Department of Rehabilitation Medicine, MOVE Research Institute Amsterdam, VU University Medical Center, Amsterdam, The Netherlands; Reade, Centre for Rehabilitation and Rheumatology, Amsterdam, The Netherlands; Department of Physical Therapy and Human Movement Sciences, Northwestern University, Chicago, IL USA; Department of Neurology, Program in Physical Therapy, Program in Occupational Therapy, Washington University School of Medicine, St Louis, MO USA; Department of Kinesiology, KU Leuven Movement Control & Neuroplasticity Research Group, Leuven, KU Belgium; Leuven Research Institute for Neuroscience & Disease (LIND), KU Leuven, Belgium; Sobell Department of Motor Neuroscience and UCLPartners Centre for Neurorehabilitation, UCL Institute of Neurology, Queen Square, London, UK; Division of Biokinesiology and Physical Therapy, University of Southern California, Los Angeles, USA

**Keywords:** Neurorehabilitation, Computational modeling, Motor control, Plasticity, Motor learning, Stroke recovery

## Abstract

Despite progress in using computational approaches to inform medicine and neuroscience in the last 30 years, there have been few attempts to model the mechanisms underlying sensorimotor rehabilitation. We argue that a fundamental understanding of neurologic recovery, and as a result accurate predictions at the individual level, will be facilitated by developing computational models of the salient neural processes, including plasticity and learning systems of the brain, and integrating them into a context specific to rehabilitation. Here, we therefore discuss *Computational Neurorehabilitation*, a newly emerging field aimed at modeling plasticity and motor learning to understand and improve movement recovery of individuals with neurologic impairment. We first explain how the emergence of robotics and wearable sensors for rehabilitation is providing data that make development and testing of such models increasingly feasible. We then review key aspects of plasticity and motor learning that such models will incorporate. We proceed by discussing how computational neurorehabilitation models relate to the current benchmark in rehabilitation modeling – regression-based, prognostic modeling. We then critically discuss the first computational neurorehabilitation models, which have primarily focused on modeling rehabilitation of the upper extremity after stroke, and show how even simple models have produced novel ideas for future investigation. Finally, we conclude with key directions for future research, anticipating that soon we will see the emergence of mechanistic models of motor recovery that are informed by clinical imaging results and driven by the actual movement content of rehabilitation therapy as well as wearable sensor-based records of daily activity.

## Background

### Nature of the problem and definition of computational neurorehabilitation

**Fig. 1:**
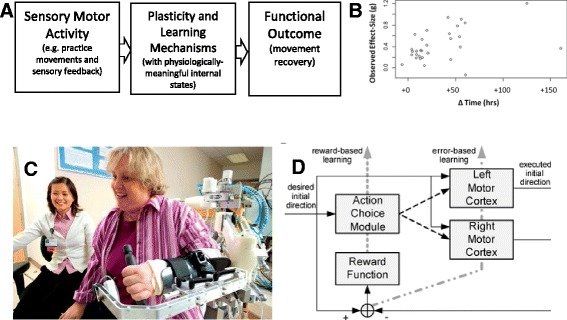
**a** General framework of computational neurorehabilitation models. Such models predict patient functional outcomes by driving computational representations of plasticity and learning with sensorimotor activity achieved in rehabilitation therapy and/or throughout the course of daily life. **b** Computational neurorehabilitation models presume that rehabilitation modulates both spontaneous biological recovery and motor learning, leading to improvements in both impaired limb motor control and compensatory movement strategies. Shown here is an estimate of the dose-response effect arising from additional therapy time, obtained by plotting effect sizes of 30 studies of upper and lower extremity rehabilitation therapy after stroke involving 1750 total participants as a function of the number of additional training hours ΔΤime. Note in this study there was no significant effect of the time the therapy was delivered after stroke (i.e. soon after stroke or in the chronic state). From [9]. Used with permission. **c** Computational neurorehabilitation models are becoming increasingly feasible in part because of a large influx of detailed kinematic data characterizing the content and outcomes of therapy, which is being obtained from robotic devices, such as Pneu-WREX shown here [218] and wearable sensors. Both individuals consented to the publication of this image. **d** Example of a computational neurorehabilitation model [112]. This model simplified neurorehabilitation dynamics by assuming that a reward-based learning mechanism determines the probabilities of using the impaired or unimpaired arms after stroke, and that a separate, error-based learning mechanism accounts for improvements in motor control through practice. The model predicts that if a patient reaches a threshold of recovery, then he or she will enter a positive cycle of using and further retraining their impaired arm through spontaneous activity in daily life, a prediction supported by data from the EXCITE clinical trial. Used with permission

Mobility-related disability arising from neurologic injury is a worldwide problem of pressing concern. For example, 16.9 million people suffer a first stroke each year, resulting in about 33 million survivors of stroke who are currently alive, making stroke one of the main causes of acquired adult disability [1]. Up to 74 % of stroke survivors worldwide require some assistance from caregivers for their basic activities of daily living (ADL) [2]. Disabling disorders such as stroke can be classified within the World Health Organization’s International Classification of Functioning, Disability, and Health (ICF) framework, which highlights the multi-tiered effect of stroke on the individual in terms of pathology (disease or diagnosis), impairment (symptoms and signs), activity limitations (disability), and participation restriction (handicap) (see Fig. 1 in refs [3, 4]). The present paper argues that mechanism-based, computational modeling of neurorehabilitation (Fig. 1) will be a valuable tool for improving rehabilitation strategies and furthering the recovery of individuals with neurologic injury at all of these levels.

At the onset, we define several terms that we will use throughout the paper, which provide a conceptual framework for computational neurorehabilitation. We will use the term “recovery” to describe improvements in movement ability over time, resulting in improvements at any of the ICF levels, regardless of how these improvements occurred. Note that this definition of recovery is similar to that used in [5, 6], but different from that proposed in [4], where the term is restricted to improvements resulting from restitution of normative biological structures and functions; we feel that “restitution” is indeed the more natural term for this more specific concept (we also found ourselves using the term “true recovery” in our discussions; others use the term “repair”.) Thus, for the purposes of this paper, we follow the nomenclature in [5, 6], in which recovery occurs through restitution, but also through compensation, which we define as use of biological structures and/or function different from those originally used before the injury to achieve a movement goal. Easy-to-understand examples of compensation are, after a stroke, using the less-affected side to perform tasks that one normally would have done with the more-affected side, or, reaching forward by leaning an abnormal amount with the trunk rather than using the usual amount of shoulder and elbow extension [7]. Note that the different modes of recovery (restitution and compensation) may occur concurrently at different levels of analysis. For example, more normal movement behavior, which appears as biomechanical restitution, may result from leveraging residual neural substrate, a form of neural compensation.

We use the terms “learning” and “plasticity” as follows (and here, we are referring to motor system learning and plasticity). If people with or without a neurologic injury train at a motor task their ability to perform the task will improve through normal skill acquisition [8]. This process of “motor learning” is dependent on plasticity, both in health and disease. In chronic stroke patients, training of appropriate tasks can therefore lead to improved function (Fig. 1b) [9]. However, the anatomy of the damage sets a limit on how much impairments, such as degraded force production capability, can be reduced in the chronic phase. Therefore functional improvements in this phase often appear to be due to learning compensation techniques [10], although targeted training may allow modest reduction in specific impairments, assessed quantitatively (e.g. [8, 11–13]). The early post-stroke period is interesting in that there are a number of injury-induced changes in the potential for plasticity, including, for example, exuberant production then activity-based pruning of new synapses [14], that may last several months, causing spontaneous biological recovery (see below) [10]. At least in animal models, motor training during this period appears to lead to a more rapid and generalized improvement in function through reduced impairment [10]. A number of therapeutic interventions under investigation can be thought of as attempts to prolong or even re-open this ‘critical period’ of plasticity, for example drugs such as fluoxetine [15, 16], non-invasive brain stimulation [14], enriched environments [17] and aerobic exercise [18]. As we will see below, so far, the interaction of this critical period with rehabilitation has not yet been well explored in computational neurorehabilitation, but it is an important target for modeling.

To make a computational analogy, recovery can be viewed as a constrained optimization problem. The amount and type of anatomical injury define the initial constraints. Unique forms of plasticity present early after injury, driven in part by experience and amenable to therapeutic interventions, act to alter the constraints, especially in early recovery. Motor learning is like the optimization itself. Motor learning that finds solutions similar to those used before the injury results in “restitution”, while motor learning that finds new solutions (which are potentially local minima) results in “compensation”. At present, it appears that intensive motor training during the early period of spontaneous biological recovery may be best suited for optimization of both surviving and new networks that results in substantial recovery of motor performance.

The idea of mathematically modeling sensorimotor recovery is not new. For example, as reviewed below, there is a rich history of research in prognostic models that take as inputs patient clinical features, baseline measurements of behavior, and/or brain imaging measurements, then predict functional outcomes at future time points using regression techniques (see reviews: [19, 20]). There are also models that have focused on altered network dynamics following injury (e.g. [21–24]), and now, the first few models that have incorporated specific aspects of rehabilitation into their dynamics (see below and related reviews [25, 26]). What is new about the computational neurorehabilitation approach is that it attempts to mathematically model the mechanisms underlying the rehabilitation process itself in order to understand the recovery of motor behavior, again via both restitution and compensation.

Specifically, we define computational neurorehabilitation models as models with three key features (Fig. 1a). Here, we describe these features in the context of sensorimotor rehabilitation, although the features can be broadened to describe other aspects of rehabilitation.

**Fig. 2:**
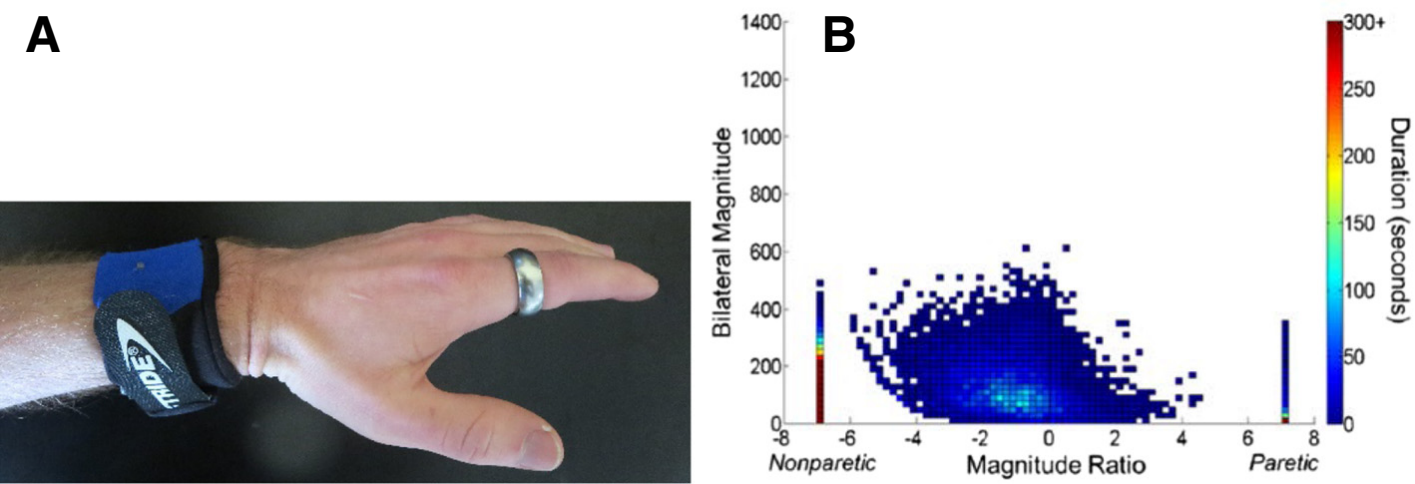
Example of wearable sensing for quantifying the daily sensorimotor activity that stimulates plasticity. **a** The Manumeter is an example of a device that monitors arm, wrist, and finger movements during daily activities [77]. The wristband is equipped with a tri-axial accelerometer to quantify movement of the arm, and thus could be used to produce data such as that shown in **b**. The wristband also contains a pair of magnetometers that quantify movement of the wrist and fingers by sensing the magnetic field changes due to a magnetic ring worn on the finger. From: [219]; Used with permission. **b** Bilateral upper limb daily activity from one individual with a stroke (ARAT score = 10) who wore a commercial accelerometer on each wrist for a 24 h period. The y-axis shows the magnitude of bilateral activity obtained by summing at each time point the vector magnitude of the acceleration of each upper limb, when each was moving over a threshold value. The x-axis shows the ratio of these two values, quantifying the contribution of each limb to the activity. Each point represents data from a one second time period throughout the day. For individuals without a stroke, these plots are symmetrical, like evergreen trees, indicating the bimanual nature of most functional activity. From [74]; Used with permission

First, such models take as input quantitative descriptions of sensorimotor activity, achieved during therapy sessions and/or throughout the day via spontaneous use of the limbs. These descriptions quantify dose and also the specific features of practice. Such data can be generated by simulations of training sessions, but are also increasingly available from actual training sessions using robotic devices (Fig. 1c) and wearable sensors (Fig. 2). The fact that computational neurorehabilitation models are driven by sensorimotor activity reflects the fundamental premise of these models, that training can improve recovery after neurologic injury. While there is considerable variability to the way this premise works out in practice, overall it is well supported by a (noisy) dose-response effect of rehabilitation therapy after stroke that has been documented in several systematic reviews [9, 27–30] (Fig. 1b). Essential to generating quantitative descriptions of the amount and quality of rehabilitation training, which can then be used as inputs in computational neurorehabilitation models, are measurements of both motor and sensory activity, which often are strongly coupled.

Second, computational neurorehabilitation models explicitly model computational mechanisms of activity-dependent plasticity. Here, we define “activity-dependent plasticity” as changes in the motor system that are caused by sensorimotor activity. Motor learning, which depends on activity-dependent plasticity, is often the basis for recovery through compensation (for example [31]) and has been the primary focus of the initial models reviewed below. Other forms of plasticity are also relevant to recovery, and these may not cause motor learning, as is clear from studies of neuronal changes early after neural injury [31]. Computational neurorehabilitation models have *internal states* that have a *biological or functional meaning* and are *dynamical* in nature (e.g. Fig. 1d). This distinguishes these models from the input/output type models that have been developed for prognostic regression (as reviewed below), or models with arbitrary internal states that are not linked to neuro-recovery mechanisms.

Third, computational neurorehabilitation models produce as outputs quantitative variables that vary with time and that relate to functional outcomes. Example outputs for computational neurorehabilitation models of arm recovery after stroke are predictions of the changes in Fugl-Meyer Motor Score, or changes in detailed quantitative measures of arm function, such as arm movement kinematics or changes in statistical patterns of the daily amount of use of the arm.

### How will computational models of neurorehabilitation be useful?

We foresee three main uses for computational models of neurorehabilitation. First, such models will provide a rigorous methodology for understanding mechanisms of recovery, that is, the biological entities and processes that implement recovery. As we survey in the next section, much is now known about various neurobiological processes important to effective rehabilitation. However, what is lacking is the integration of the processes, which operate at widely different spatial and temporal scales. Developing computational models of rehabilitation will force researchers to make these processes and their dynamic interactions more concrete. We thus believe that computational neurorehabilitation will become essential for providing frameworks to organize a diverse and growing body of data. Such multi-level computational approaches are already playing important roles in fields such as HIV [32, 33] and cancer treatment [34].

The second use of these models will be to aid in designing new clinical experiments. Currently, optimization of rehabilitation proceeds slowly in a trial-and-error fashion that is overly dependent on large and very expensive clinical trials that include multiple testing points for each participant. As in engineering design, a mechanistic, mathematical model of the system of interest will allow the effect of variations in rehabilitative parameters to be simulated, allowing a means to design potentially more effective experiments. Initial examples of this approach have already been demonstrated in the related field of motor adaptation, in which computational models of adaptation have been used to conceptualize behavioral environments that accelerate an individual’s ability to learn, e.g., [35], although adaptation is a somewhat limited type of motor learning to study for rehabilitation purposes. Nevertheless, we expect, by analogy, that new mathematical models of behavioral interventions relevant for rehabilitation will provide a means to conceptualize and design studies that can potentially enhance recovery. Use of models will also help guide collection of the types of data that can answer important mechanistic questions.

The third use of computational neurorehabilitation models relates to the second but extends it, and is to optimize therapy selection for individual patients, in terms of dosage, timing, scheduling, and content. How much therapy should patient X receive? At what time and according to what schedule should this therapy be provided? What movements should he or she practice with what sort of instructions and feedback? Currently, treatment modality and dose are mainly determined based on clinical opinion or historical precedent. In some cases, data from clinical trials influence these choices, but these data reflect averages from large groups of patients. Computational models of stroke recovery will enhance precision medicine and improve stratified trials by allowing better selection of patients for specific evidence-based therapies as well as optimizing the dosage of such therapies. For example, based on current knowledge about the predictive value of the shoulder-abduction-finger-extension (SAFE) model for the upper paretic limb within 72 h post stroke, a prognostic algorithm for selecting evidence based therapies was recently developed as a smartphone app [36]. We expect in the future, that these computerized prognostic algorithms for improving task-specific treatments may be further optimized by additional information from neuroimaging [37] and more sensitive information from kinematic assays about quality of motor performance post stroke [8]. Computational neurorehabilitation models will further enhance these efforts by incorporating explicit representations of plasticity and learning mechanisms, potentially improving predictive capability.

The idea for this review resulted from a small colloquium on computational neurorehabilitation sponsored by the Borchard Foundation in July 2013 in France. At this meeting, researchers from complementary disciplines, including neuroscience, movement science, rehabilitation, neurology, robotics, and engineering, overviewed the latest data available to develop such mechanistic models, and critically evaluated several first modeling attempts that are available. Based on our interactions, we argue that principle guided neurologic recovery and, as a result, accurate predictions at the individual level will be facilitated if algorithmic computational models of learning behavior, and eventually of fine-level neural processes, are developed and integrated into a context specific to rehabilitation. To develop this argument, we first review here model elements for computational neurorehabilitation, and then review the current gold standard in rehabilitation modeling – prognostic regression models (Table 1). We finally review several initial computational neurorehabilitation models, before concluding by summarizing the state of the field and identifying needed directions for future research.

**Table 1:** Organization of this review

Introduction	
Nature of the problem and definition of computational neurorehabilitation	
How will computational models of neurorehabilitation be useful?	
Review	
I. Model elements for computational neurorehabilitation	
A. Inputs: Sensorimotor Activity	
B. Innards: Modeling activity-dependent plasticity	
C. Outputs: Functional outcomes and kinematics	
II. The Current Modeling Benchmark: Prognostic Regression Models	
A. Predicting outcome post stroke with baseline behavioral measures	
B. Predicting outcome post-stroke with brain imaging measures	
C. Predicting treatment effects	
III. Computational neurorehabilitation models	
A. Reaching the threshold for recovery in bilateral hand use	
B. Recovering from weakness via reinforcement learning	
C. Robot assistance, retention, and learning predicts recovery	
D. Understanding interactions between function and use	
E. Modeling the effect of assistance-as-needed	
F. Patient-trainer dynamics as an optimization	
Conclusions	

## Review

### Model elements for computational neurorehabilitation

This section reviews the key elements needed to construct a computational neurorehabilitation model, which are A) a quantitative description of the sensorimotor activity that the patient experiences; B) a computational model of the plasticity mediating recovery; and C) a quantitative description of the patient’s behavioral outcomes. To provide a specific context for the discussion, we again concentrate on strokes affecting motor control of the upper extremity, as much of the initial work in computational neurorehabilitation is being done in this area.

#### Inputs: sensorimotor activity

Modeling activity-dependent plasticity requires a quantitative description of activity that stimulates plasticity. Historically, sensorimotor activity during neurorehabilitation has been characterized in research studies and clinical practice primarily by the amount of time spent in assigned therapy sessions [9, 27]. It is also possible to simulate training sessions, in order to derive theoretical inputs for models, as has been done for most initial models described below. However, one reason that computational neurorehabilitation models have the potential to soon become much more elaborate and powerful is that researchers are beginning to quantify more precisely the sensorimotor activity that a patient experiences. There has been increased interest in quantitative, observational studies, and in new sensing technologies, including robotics and wearable sensors.

##### Observational studies of rehabilitation therapy

Recent observational studies found that although stroke patients spend approximately 47 min in occupational therapy each day in early rehabilitation, only 4–11 min of this time is focused on upper extremity rehabilitation [38, 39]. Distinguishing between total therapy time and active movement time is essential for accurately driving computational neurorehabilitation models. Another fundamental question that was only recently answered is “How many practice movements are typically made during rehabilitation therapy?” For the upper extremity after stroke, a study of 162 rehabilitation sessions in seven sites yielded an average of 32 functionally oriented movements [40]. Notably, this number of movements per session is an order of magnitude less than the number of movements per session that has been shown to induce motor plasticity in animal models [40]. There is evidence that upper extremity interventions can be designed to provide such a larger number of repetitions without increasing therapy duration [41–43].

##### Quantification of sensorimotor activity during therapy

Use of robotics and sensor-based therapies, including virtual rehabilitation [44, 45] and exergaming/serious games [46], has grown rapidly in both rehabilitation research and practice in the last 20 years [47–52] although the overall percentage of clinics using these new technologies is still relatively low [53]. The primary motivation for developing these technologies is to provide a greater dose of therapy, but an important secondary benefit relevant to computational neurorehabilitation models is that these technologies can continuously measure the sensorimotor activity of the patient during therapy. For example, a robotic device that assists in therapy of the upper extremity (Fig. 1c) can measure the forces and motions that a patient experiences during training, providing insight into both the motor commands and intrinsic biological feedback that result from those commands. Such a device can also record any external, augmented sensory feedback – visual, audio, and haptic – that the patient experiences during training, since this feedback is provided by the device itself (assuming the therapist is not also providing feedback). Quantifying feedback content as well as movement itself is important because feedback powerfully modulates motor learning [54] and rehabilitation [55].

Examples of the type of data available from robotic and sensor-based therapy devices include the number of movements made and the trajectories achieved while making these movements. Other key variables relate to kinetics, such as the forces applied by the robot to the patient [56, 57] or by the patient to the robot [58], or amount of positive and negative work done on the patient during therapy with the device [59, 60]. Isolated sensors can also quantify the physical interaction forces and motions that therapists apply during hands-on therapy [61]. Such biomechanical measurements can be combined with measures of Electromyography (EMG) to generate estimates of muscle activity during training, and, increasingly, brain imaging techniques, including Electroencephalography (EEG) [62], Near-Infrared Spectroscopy (NIRS) [63], and functional Magnetic Resonance Imaging (fMRI) [64], to provide insight into brain activity during training.

##### Quantification of sensorimotor activity during daily activities

In rehabilitation research there has been an increasing recognition that the sensorimotor activity experienced during therapy is only one part of the total sensorimotor activity that drives recovery, or, put another way, that daily use of the arm likely also plays a major role in aiding recovery [65]. Again, new technologies, in this case wearable sensors, are now making it possible to quantify this daily activity beyond how it was done in the past [66, 67], i.e. through patient self-report scales such as the Motor Activity Log [68]. The primary approach being used so far for the upper extremity is wrist accelerometry, in which a three-axis accelerometer is embedded in a wristband [69–72].

Wrist accelerometry is typically used to detect the amount of time spent moving the arm using a thresholding approach [73]. If sensors are worn on both arms, the amount of bimanual activity can be quantified, and the activity of the stroke-impaired arm can be compared to that of the less affected arm [74]. Indeed most human motor activity seems to be bimanual in nature [74, 75], which has implications for how computational neurorehabilitation models should be structured. New wearable sensing approaches are making it possible to non-obtrusively quantify finger and hand activity as well as gross arm movement during daily life [76, 77].

#### Innards: modeling activity-dependent plasticity

Given a quantitative description of sensorimotor activity during stroke recovery, a computational neurorehabilitation model uses this description to drive a mathematical model of activity-dependent plasticity mechanisms. Here, we briefly overview two types of activity-dependent plasticity that will play a key role in computational neurorehabilitation models – one related to spontaneous biological recovery, and one related to motor learning. For reviews see [78–80]. Note that for ease of presentation we speak of plasticity and learning rules as if they were independent form the model structure, but for most models they will not be. The model will need to consider how the necessary anatomical and functional structures support learning and plasticity, regardless of the abstraction level of the model.

##### Spontaneous biological recovery and activity-dependent plasticity

Many initial performance changes after stroke are attributed to “spontaneous biological recovery”, a term that implies that this recovery is automatic, although spontaneous biological recovery is almost certainly modulated by and requires behavior for maximal expression [10, 14]. Animal models indicate that spontaneous biological recovery is aided by a significantly altered tissue microenvironment triggered by the injury, in which, for example, a different profile of genes is expressed compared to during normal motor learning [31]. Spontaneous biological recovery also involves both reduction of the ischemic penumbra and brain reorganization in areas both near the lesion and farther away [81]. Spontaneous biological recovery is maximally expressed in the first several weeks post-stroke, and tapers off over months [10, 14, 20, 82]. Brain reorganization processes underlying this spontaneous recovery are thought to be driven by homeostatic mechanisms, Hebbian-like processes driven by long-term potentiation (LTP) [83, 84], as well as spine, dendritic and axonal forms of structural plasticity.

Soon after stroke, abnormal cortical patterns of excitation and inhibition occur both near [84–87]) and far from the lesion [88]. Homeostatic plasticity, which is ubiquitous in the brain, acts to maintain desired firing rates and patterns [81]. After a lesion, because of the loss of interneuronal connections, the activities of neurons neighboring the lesions, or neurons previously connected to neurons within the lesion, are affected. Homeostatic plasticity may be crucial for network recovery, as measured by re-establishment of lesion-affected inputs [89]. In addition, sensorimotor activity might modulate this homeostatic plasticity, which is of importance for computational neurorehabilitation models, as it is one example of how sensorimotor activity appears to modulate spontaneous recovery [6, 31, 89, 90].

LTP, LTD and neural structural plasticity such as dendritic and axonal sprouting, are also modulated by sensorimotor activity, and also change as a function of time. Following stroke, some features of brain function revert to those seen at an early stage of development, with the subsequent process of “recovery recapitulating ontogeny” [91], but there is also a distinct, age-related pattern of gene expression, a “recovery transcriptome” [92]. Genetic changes in the perilesional area allow for a window of increased plasticity that makes it easier for the perilesional neurons to modify existing connections and form new ones in response to sensorimotor activity [81]. Increased LTP may also potentially lead to maladaptive plasticity and poor cortical reorganization if existing inputs are further strengthened at the expense of the reemergence of weak afferent synapses [89]. In summary, underlying mechanisms assumed to contribute to the non-linear time course of recovery of movement in the first 3 months after stroke presumably reflect the interaction between a period of heightened plasticity mechanisms, occurring in a limited time window, and sensorimotor activity [81, 93–95]. A practical implication is that, when new patterns of movement that are a consequence of specific combinations of muscle weakness (e.g. increased trunk flexion and abduction of the shoulder during reaching) are attempted repeatedly during this period of heightened plasticity, they may become the new ‘norm’ – hence patients get stuck in local minima. Further, use of the less-impaired arm may subvert the heightened plasticity of the stroke-affected hemisphere, preventing it from improving the paretic arm function [14].

There are as yet few computational models of spontaneous biological recovery, much less of the interaction between spontaneous biological recovery and sensorimotor activity. Computational models of the effects of stroke to date have primarily focused on the network effects of deleting cells or of altered connectivity. For example, one early model used a difference-of-Gaussians connectivity pattern to explain rapid changes in the size of cellular receptive fields after stroke lesions [96, 97]. Other models have studied interhemispheric effects of lesions [23, 24], and used connectome data to model brain regions as graphical network nodes, evaluating the effects of node deletion on network dynamics [98–100]. One of the first models to study the effects of network changes on movement kinematics evaluated the effect of lesion size on post-stroke reach variability using cortical cells that were tuned to preferred reach directions, but did not simulate plastic processes after lesion [101]. A recent model studied the interaction between homeostatic plasticity and Hebbian-plasticity after stroke in the somatosensory cortex, and suggests that after a lesion, a delay preceding rehabilitation would allow a return of homeostatically-determined desired firing in cells neighboring the lesions, and thus may allow a faster network recovery in the rehabilitation training compared to no delay [89]. It will be increasingly important to compare models that incorporate spontaneous biological recovery mechanisms to ones that do not, to determine how modeling these phenomena improves explanatory power. New analytical approaches to examine structural and functional connectivity within well-defined macroscopic brain networks, as briefly reviewed in Section II C below, will increasingly play a role, and will integrate plasticity rules with the necessary anatomical and functional structures.

##### Motor learning

Although some aspects of neural reorganization involved in spontaneous recovery arise because of the unique biological state caused by injury, other aspects of neural reorganization that contribute to recovery relate to normal motor learning mechanisms [78, 79, 102]. It has been argued, in fact, that much of the recovery seen during the chronic phase of stroke is due to compensation, as defined in the introduction, which is enabled by motor learning [31]. In this section, we briefly survey several models and features of motor learning relevant to computational neurorehabilitation models.

##### Forms of learning

There is a long-history of research in artificial intelligence linking different types of feedback to three forms of learning: unsupervised (or Hebbian) learning, supervised learning, and reinforcement learning, or, more simply paraphrased, learning features and representations, learning from errors, and learning from rewards [103]. To learn a motor task, the learner needs feedback, exteroceptive, interoceptive, or both; as a result, in addition to unsupervised learning, supervised or reinforcement forms of learning are implicated in rehabilitation. Indeed, feedback, including the content and frequency of feedback, is known to modulate learning and rehabilitation efficacy [54, 55]. Different forms of learning have been associated with different biological substrates, although there is not a one-to-one mapping and the picture is still unfolding [103]. For example, a form of unsupervised learning is LTP in pyramidal neurons [104]. Supervised learning-like plasticity has been found to occur in the cerebellum [105, 106]. Some forms of reinforcement learning depend to a large extent on the dopaminergic system [107, 108], with the basal ganglia [109] and in particular the nucleus accumbens [110] also playing roles. Note that supervised learning is linked to the concept of Knowledge of Performance (KP) and reinforcement learning to the concept of Knowledge of Results (KR) in the motor learning and rehabilitation literature [54], although KP and KR likely also both act as reward signals for reinforcement learning, thus blurring this distinction.

Unsupervised learning is related to the concept of use-dependent learning, which refers to the phenomenon that the motor system can modify its performance through pure repetition of movements, without external feedback as to the success or failure of the movement [103, 111]. Several initial models of network dynamics after stroke incorporate unsupervised learning (see review [25]). Unsupervised learning, together with homeostatic plasticity, likely plays a role in map and neural reorganization post-stroke, and presumably in decreasing movement variability and thereby improving functional performance [89, 112].

##### Supervised learning and arm adaptation

A large number of studies of motor learning in the last 20 years have focused on elucidating aspects of supervised learning by observing the adaptation of arm movements in visuomotor rotations or force fields produced by robotic interfaces. It is still unclear how meaningful such studies of motor adaptation are for stroke rehabilitation, but they have inspired at least two innovative rehabilitation paradigms – error augmentation [113] and split-belt treadmill adaptation [114]. Such studies have also generated some of the first and now fairly sophisticated mathematical models of motor learning, and thus serve as an example for how the development of computational models in stroke movement rehabilitation might proceed. Further, adaptation studies are relevant because individuals with a neurologic injury can adapt to predictable perturbations (e.g. [115, 116]), and likely continue to use motor adaptation to recalibrate limb systems in daily life (e.g. when they put on new shoes of a different weight, enter a swimming pool, or experience muscle fatigue [117]).

Adaptation studies have shown that humans interact with novel environments by minimizing error (e) relative to the planned movement, and effort (u) [118, 119]. This can be modeled as the minimization of the cost function.

1 $$ \mathrm{V}={\upalpha \mathrm{e}}^2+{\upbeta \mathrm{u}}^2,\upalpha, \upbeta >0. $$

A key recent result is that a simple neural algorithm, which is a “sunken-v”, muscle-specific activation update law that relates the error experienced in muscle coordinates to the change in in muscle activation on the next movement trial, implements this minimization, while simultaneously shaping arm impedance to the task at hand [118–120]. Another factor involved in movement generation is that subjects tend to minimize time to complete an action, which stands in tradeoff with the required effort [121].

##### Time-scales

Learning occurs at multiple time scales as short as 10s of seconds [122, 123] and as long as several years [54]. Multiple time-scales are also evident in the learning-performance distinction [102, 124], which will impact how models of recovery are structured. This sort of multiple time-constant dynamic also characterizes a broad range of motor learning literature encompassing a broader variety of tasks. Motor adaptation studies also shed light on multiple time scales, as motor adaptation occurs via simultaneous update of a fast process, which contributes to fast initial learning, and a slow process, which correlates with long-term retention [122]; these processes appear to be organized in parallel [125]. Linear models with two time-constants implemented using a state space representation can account for a range of data on motor adaptation such as anterograde interference, spontaneous recovery, and savings under some conditions. Addition of non-linearities in the model allow for multiple adaptation and savings after washout [125, 126].

Whereas behavioral observations suggest that at least two learning processes are involved in adaptation, it is unclear how many distinct memories the brain actually updates. In addition, it is unclear whether these putative multiple motor memories reside within a single neural system that contains a distribution of possible timescales, or in qualitatively distinguishable neural systems. A recent study addressed these issues using a model-based fMRI approach [127]. The behavioral data of subjects adapting to two opposing visuo-motor perturbations were first used to derive a large number of possible memory “states”, each with different dynamics, which were then correlated with neural activities. Regional specificity to timescales were identified. In particular, the activity in inferior parietal region and in the anterior-medial cerebellum was associated with memories for intermediate and long timescales, respectively. A sparse singular value decomposition analysis of variability in specificities to timescales over the brain identified four components, two fast, one middle, and one slow, each associated with different brain networks. Then, a multivariate decoding analysis showed that activity patterns in the anterior-medial cerebellum progressively represented the two rotations. These results thus support the existence of brain regions associated with multiple timescales in adaptation and a role of the cerebellum in storing multiple internal models.

Note that these multiple-time constant models assume error-based learning mechanisms. A recent summary of behavioral evidence concluded that while there are at least two components of motor adaptation in response to perturbations, they cannot be fully characterized by first order processes driven by error. For example, the slow process is implicit and learns form errors, while the fast process is explicit and is sensitive to success and failure, among other key differences [128]. The evidence for reward-based and use-dependent mechanisms in motor adaptation suggest they operate at multiple time constants as well, and are likely to be of more relevance to restitution rather than compensation [129–131].

Such state-space models can account for short-term motor adaptation as well as multiple task learning and the contextual interference effect in post-stroke individuals [132, 133]. At least one initial computational neurorehabilitation model successfully used state-space models inspired by supervised learning data [56]. In addition, robotically amplifying errors can help stroke patients eliminate steady state directional reaching errors [116]. Further, training with amplified errors may increase arm movement recovery after chronic stroke [113]. The beneficial effects of sensory augmentation may be due to the larger error available to the brain for perception and for learning. A recent study however suggests that augmenting errors can decrease motivation in a way that persists beyond the experience of the augmented errors [134], and motivation plays a key role in neurorehabilitation [112].

##### Reinforcement learning

However, as mentioned above, the relevance of mechanisms of supervised learning of force fields and rotations to rehabilitation is limited, in part because rehabilitation, like motor skill learning, appears to be characterized by gradual improvement without a clear directional error signal, as when one tries to perform a fast movement or loses balance [130]. This type of learning appears to be better characterized by reinforcement learning. Reinforcement learning theory [135] provides a framework for learning a “control policy” that maps states of the world to the actions that the agent should take in those states to maximize expected future rewards (or equivalently minimize future cost such as effort). Crucial to reward-based learning is the concept of exploration or search, which is necessary because there is no teacher. Instead, the learner must learn by trial and error. Compared to supervised learning, much less is known about how humans make use of reinforcement learning in learning motor behaviors; the issue has been explored to a larger extent in decision making. However, as we describe below, this search metaphor has been used successfully to simulate stroke rehabilitation, and replicate several key behavioral recovery observations [112, 136].

Note that both supervised and reinforcement learning likely operate simultaneously as both error and reward feedback are often available [137]. For instance for fast reaching to targets by unimpaired subjects, it has been shown that different time constants of learning, and forgetting, may be associated with supervised and reinforcement learning [130]. In rehabilitation therapy, receiving error feedback from a therapist can be rewarding and reinforces behavior. In the absence of external feedback, the learner still has access to intrinsic feedback and this can strongly promote self-learning. Thus, what is presumed to be unsupervised learning can be instead reinforcement learning driven by self-generated feedback. Also, it is the self-generated feedback that the patient needs to rely on when returning to his or her home environment. Accordingly, whenever external feedback is provided, it is important not to become too dependent on this source of augmented information by gradually weaning the learner from external feedback during practice, i.e. to learn to rely on self-generated feedback [137, 138].

Humans do not always appear to minimize error or maximize future rewards, however. In some instances, humans tend to perform a motor task by using the same strategy as they had used in previous trials, even if they had previously experienced a strategy using much less effort [139–142]. This suggests that rather than attempting global minimization of effort, the sensorimotor system might rather repeat a strategy that it knows will achieve the goal, a finding with implications for modeling use of compensatory movements by stroke patients.

##### Smoothness, generalization, and synergies

Several additional key aspects of motor learning that computational neurorehabilitation models will ultimately need to account for are the importance of sub-movements, generalization, and suppression of undesirable synergies. It is well known that the movements of individuals with stroke exhibit decreased smoothness. Variations in smoothness can be modeled as arising from patterns of stereotypical sub-movements, which may be neural “building blocks” that rehabilitation training must reassemble [143].

Generalization refers to the concept that training on one task can improve performance on other tasks. Patterns of generalization are complex, in that generalization has been found to be limited in some conditions [143], but rather broad in others [54]. For example, after training to reach in one direction with a planar robotic perturbation, there is little transfer to other directions [144], but relatively broad generalization across certain arm postures [145]. The concept that motor generalization is rather limited has helped drive a strong focus on task-specific training after stroke [146]. However, a key qualifier of this concept is that the organization of practice may determine how much generalization occurs. If one trains one specific task or task variant, there may be little transfer. However if many task variants are practiced, transfer will likely be larger. This is called the variability of practice hypothesis [147], and has clear relevance for computational neurorehabilitation models.

Finally, motor learning not only involves building new action patterns but also suppressing or modulating pre-existing patterns or synergies. This is clear in bimanual skill learning in which the learner gradually overcomes the effect of pre-existing preferred coordination modes (such as in-phase and anti-phase patterns) that are part of the intrinsic dynamics of the system in order to acquire new coordination modes (such as less intrinsic, relative phase patterns) [75]. Like any motor learner, individuals with a stroke may be more constrained by preferred coordination modes and/or by basic synergies that need to be overcome to develop skill. Similarly, previously acquired coordination modes can hamper the acquisition of new coordination modes, a phenomenon called negative transfer [148]. Further, neural damage itself may fundamentally constrain the solutions possible for motor learning.

#### Outputs: functional outcomes and kinematics

Currently, in rehabilitation, behavior is usually described with relatively coarse scales, which often sum scores of performance on many tasks, and are typically taken at widely spaced time points. Ideally, computational neurorehabilitation models will bridge the causal link between network plasticity and behavioral changes, which will require higher resolution measurements of behavior at many repeated time points.

Note that data sets that evaluate outcomes differ from the data sets discussed in Section I A in that they quantify how much and how well a patient can move, rather than the total amount and features of rehabilitation training activity. There can be overlap between the two data sets, however, in that measurements made during training can be used to assess movement outcomes, and measurements made of movement during daily life can be used to quantify both training inputs (inasmuch as daily movement serves a training function), as well as serve as a way to quantify outcomes. For instance, kinematic measurements from a robotic rehabilitation device obtained during the course of robotic therapy have been shown to predict standard functional outcomes, without the need for dedicated assessment procedures [149].

Higher resolution outcomes data are becoming available through detailed kinematic studies of upper extremity movement in stroke recovery. In one study that serves as an example, patients with active proximal and distal limb movement within the first 2 weeks after stroke participating in the VECTORS trial were studied with kinematics and electromyography, identifying deficits in movement accuracy, reduced muscle efficiency, delayed muscle onsets, and a reduced ability to modulate muscle activity [150, 151]. Within the first 3 months after stroke, muscle onset times and percentage of muscle capabilities were similar to a neurologically-intact control group, but deficits in the ability to modulate muscle activity remained [151], including an inability to efficiently open and close the fingers on a target object [152, 153]. No computational neurorehabilitation models have yet to our knowledge attempted to model these outcomes.

Other longitudinal movement data from multiple labs around the world are accumulating [7, 154, 155]. For example, a recent kinematic study with intensive repeated measurements in the first months post stroke used principal components analysis to show that individuals with stroke learn to dissociate shoulder and elbow movements mainly in the early phase post-stroke, but do not achieve fully dissociated movements even at 26 weeks [7]. Likewise, recovery in smoothness in reaching and hand aperture was mainly predicted by progress of time alone and almost plateaued within the first 8 weeks post stroke [156]. Again, no models that incorporate plasticity mechanisms have yet attempted to model these findings.

Ideally, motion capture data sets would include the effect of different interventions. For example, there is an ongoing debate on the issue of whether recovery of functional movement is best achieved through restitution (such as reaching with normal kinematics) or compensation (such as using the less affected extremity or leaning forward with the trunk) [4, 157, 158]. At the present time, there are only small amounts of movement data collected pre- and post-intervention to address this issue. In a pilot trial of intensive, progressive, task-specific upper extremity training for people with stroke [41], kinematic and kinetic movement data were examined pre- and post-intervention to examine how movement changed [159]. The results suggest that recovery of function via restitution versus compensation is not an all-or-none phenomenon, but varies within and across individuals. All patients demonstrated improvements in function on clinical scales. In contrast, some movement variables in some subjects indicated restitution of normal movement patterns, while other variables in the same or different subjects indicated the adoption of compensatory movement patterns [159].

Just as wearable sensors will drive computational neurorehabilitation models with data from self-training of the arm during home exercise or daily life, they will provide the descriptors of movement recovery that the models seeks to predict. Such sensors will provide data at a much finer temporal resolution than previous clinical data, which typically are obtained only at baseline, post-intervention, and at one or two follow-ups. This fact, along with the fact that the sensors provide kinematic data, will facilitate simulation of neural networks controlling movement recovery. Such technology-based measurements are also being found to map well to clinical outcome scales [149, 160–162]. Thus, these measures have validity in terms of established clinical measures, while enhancing the interpretation of these measures, facilitating more fine-grain modeling, and developing new measures. Further, sensor-based measures may catch improvements or differences in behavior when clinical assessment suffers from lack of resolution or floor/ceiling effects, e.g. [163]. Again, we are at a propitious time for computational neurorehabilitation because of the rapid rise of new wearable sensing technologies, the data from which can be used to quantify functional outcomes important to patients in clinicians.

### The current modeling benchmark: prognostic regression models

There is a rich body of work on statistical modeling of stroke recovery using regression models. A primary motivation for this work has been to develop prognostic models that support clinical decision making with regards to early stroke management, rehabilitation goals, and discharge planning [20]. Such models take a “black-box” approach, seeking to identify the mapping between inputs, such as behavioral status and brain structure and function soon after stroke, and patient outcomes, such as long-term functional recovery. Such models can be driven solely by behavioral data combined with clinical descriptors of patients, by neurophysiological data (e.g. EMG, (f)MRI and Transcranial Magnetic Stimulation), or by both. These models usually do not have a priori hypotheses regarding the basis of this mapping in specific plasticity mechanisms, but they form a key benchmark against which the prognostic power of computational neurorehabilitation for clinical decision-making must be tested. That is, a key question is: “Will adding mechanistic details provide additional predictive power useful for clinical practice?” Accordingly, this section provides a brief overview of prognostic regression models.

#### Predicting outcome post stroke with baseline behavioral measures

Prospective cohort studies suggest that 33 to 66 % of stroke patients with a paretic upper limb do not show any recovery in upper limb function 6 months after stroke [164, 165]. In contrast, depending on the outcome measures used, 5 to 20 % will achieve full recovery of activities at 6 months. Multivariate regression models that are aimed to predict these outcomes are based on identifying variables (i.e. “markers” or “predictors”), usually measured at baseline, that are linearly or logistically associated with patient outcome at a later time post stroke. Baseline behavior markers that have been found to be useful in such models are voluntary movement ability measured at key joints, initial gross severity of stroke, initial disability, initial severity of motor deficits (e.g. Fugl-Meyer motor scores), and initial kinematics of reaching movements [7].

Two independently conducted prospective cohort studies showed that 98 % of individuals who preserve some voluntary finger extension and some shoulder abduction when assessed within the first 72 h post stroke regain some function at 6 months [20, 166, 167]. However, only 25 % of patients without voluntary control at 72 h regained some function at 6 months. The small proportion of false positives (≈2 %) and relatively large proportion of false negatives (≈25 %) in the SAFE model (Shoulder Abduction Finger Extension) [167] suggests that this clinical model may be too pessimistic in identifying patients who are likely not to recover meaningful function. The preservation or early return of some finger extension most likely reflects the necessity of some fibers of the corticospinal tract system in the affected hemisphere to remain intact in order to activate muscles of the forearm and hand [166, 168].

Numerous studies have also shown that the initial overall severity of stroke measured within 72 h after stroke onset, for example by using the NIH Stroke Scale [169] and initial disability, for example measured with the Barthel Index [170], are highly associated with the final outcome at 6 months measured with the NIH Stroke Scale, Barthel Index, or Functional Independence Measure. Retesting the Barthel Index at regular intervals significantly improved the model accuracy. These findings indicate that the timing of clinical assessment post stroke is an important factor that defines the accuracy of predicting final outcome [20].

**Fig. 3:**
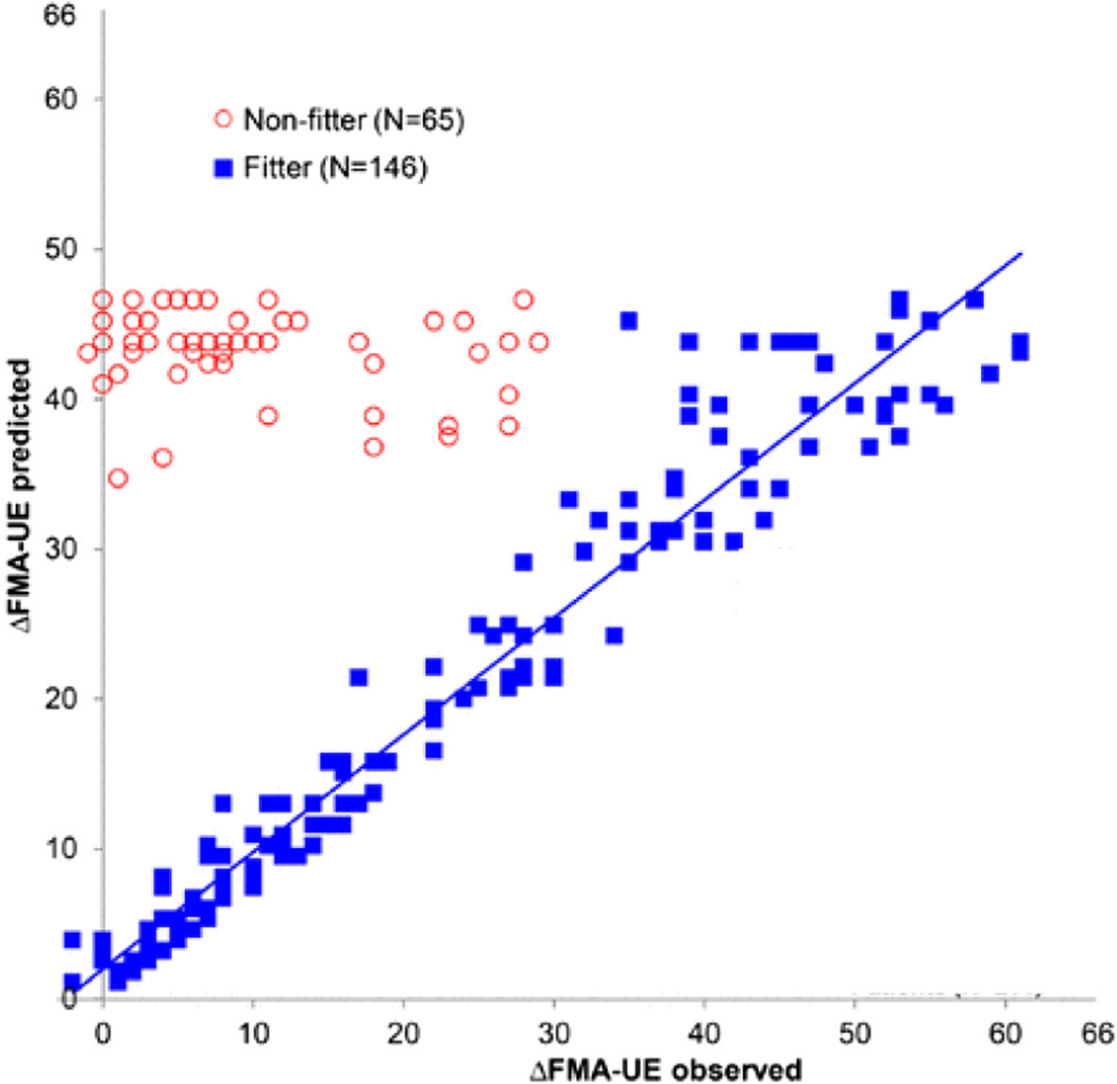
Example of the predictive power of a prognostic regression model, the proportional recovery model [171] (see Eq. 2). The model accurately predicts the change in upper extremity Fugl-Meyer score from 2 days to 3 months post stroke for 70–80 % of the patients, who all received rehabilitation. The subgroup of patients who did not fit the model experienced less recovery than predicted. To our knowledge, there are no computational rehabilitation models that can predict which patients will fit this prognostic regression model, or explain the variance in those who do not. Modified from [173]; Used with permission

The upper extremity Fugl-Meyer score, an assessment of arm movement ability in which various test movements are scored on a three point ordinal scale and then the component scores are summed to form a single number, also predicts motor recovery. In 2008, it was shown that approximately 70–80 % of stroke patients follow a “proportional recovery rule”, recovering about 70 % of their maximal potential recovery at 3 months based on the initial Fugl-Meyer motor scale [171–173] (Fig. 3). This can be expressed as:

2 $$ \Delta \mathrm{F}\mathrm{M}\approx 0.7\cdotp \left({66\ \hbox{-}\ \mathrm{F}\mathrm{M}}_{\mathrm{initial}}\right)+0.4 $$

where ΔFM is the predicted change in upper extremity Fugl-Meyer score at 6 months and FM_initial_ is the score measured within 72 h. This rules suggests either that 1) patients receive a dose of therapy proportional to their impairment, 2) some basal amount of rehabilitation is required for spontaneous recovery, or 3) current rehabilitation does not strongly modulate impairment recovery [10], hypotheses that could be explored with computational neurorehabilitation models. In addition, at lower (i.e., more severe) values of FM_initial_, this relationship is not as accurate, with approximately 20–30 % of patients showing a much smaller ΔFM than that predicted by the model [171–173]. Outliers not fitting the line of proportional fixed recovery suffered from more severe hemiparesis and multimodal impairments such as sensory deficits and neglect [133].

Note this equation is a first order equation in FM. Thus, proportional recovery appears to describe a rule of spontaneous biological recovery that follows first order dynamics [10]. In this framework, any early functional improvement that ensues is mainly happening via reductions in neurological impairment (i.e., restitution). Regression analyses of change scores in early started intensive repeated measurements post-stroke have shown that this time-window of spontaneous neurological recovery appears to be restricted to the first 3 months post stroke [174]. Beyond this time, improvements in function appear to be mainly driven by compensation strategies in which patients learn to use their intact end-effectors to optimize motor performance [7, 79, 156, 175]. See also reviews about definitions, terminology [119] and phenomenology [6] of stroke recovery.

A recent development in prognostic modeling is the use of multiple input variables in non-linear regression models such as neural networks. Specifically, a set of kinematic measures assessed with a robotic device, then mapped through a nonlinear mapping algorithm, predicted clinical outcomes after stroke with higher precision than baseline clinical measures alone [162]. This 30 min assessment asked patients to reach in the horizontal plane to visual targets, to draw circles, and to move against robotic resistance. Metrics included deviation from a straight line, aim, average speed, peak speed, movement duration, smoothness of movement, and features of the sub-movements of movement trajectories, including number, duration, overlap, peak, interval, and skewness of sub-movements. Model performance plateaued when using about eight of these features in the predictive model; that is, additional features did not improve prediction, presumably because of high correlations between some of these features. The nonlinearity of the model was essential to its improved predictive power, which suggests that successful computational neurorehabilitation models will incorporate nonlinearities as well. Note that this model, like “classical” regression models, is purely statistical, and does not explicitly model brain reorganization or learning following stroke.

#### Predicting outcome post-stroke with brain imaging measures

Another way to look at proportional recovery is that anatomical damage sets a limit on the extent of possible recovery by restitution and therapy takes advantage of plasticity mechanisms (whether enhanced during the early period of spontaneous biological recovery or normal in the chronic phase) to help patients achieve the maximum recovery possible. Predictive models thus need to include neuro-anatomical variables [176]. However, given that level of initial severity is the best predictor of final outcome, we need to ask whether brain imaging data can improve model performance. In other words, we are interested in the factors that determine recovery over and above those that cause impairment, as these are different things [172].

Residual structural and functional architecture after stroke can also be used to estimate clinical outcome. Diffusion tensor imaging (DTI) is able to assess integrity of white matter tracts and when measured within 3 weeks of subcortical stroke, corticospinal tract (CST) integrity correlates with both initial and 6 month upper limb impairment [177]. In a separate study, damage to the CST at the posterior limb of the internal capsule 12 h post-stroke correlated well with motor impairment at 30 and 90 days [178]. Stinear and colleagues have proposed an algorithm for sequentially combining simple clinical, TMS, and DTI measures to predict upper limb function [168]. The PREP (Predicting REcovery Potential) algorithm was tested in a sample of 40 sub-acute stroke patients and performed well in predicting motor function based on Action Research Arm Test scores at 12 weeks post-stroke. The performance of DTI in this setting should be improved by making the tracts specific to particular functions e.g. upper limb [179] and developing ways for the assessment of tract integrity to be done in a standardised [180] and automatic [181] manner.

Corticospinal tract integrity correlates with initial upper limb severity, which explains why it often correlates with final outcome. However, it remains unclear whether it can explain outcomes over and above initial impairment. The role of intact cortical regions in supporting motor recovery is unknown, but has been explored in language recovery. Predicting language outcome and recovery after stroke (PLORAS) uses the whole structural brain scan from which voxel-wise estimates of the likelihood of damaged tissue are derived [182]. This ‘lesion-map’ for each patient is added to (i) time since stroke and (ii) a detailed assessment of various language capabilities. Using a machine learning approach, a new subject's lesion image is compared with those from all the other patients already in the database to find one with a similar lesion. The language scores for all the similar patients are plotted over time, enabling the time course of recovery for the new patient to be estimated [183]. The potential for such an approach extends to many domains including motor and cognitive outcomes. Using this type of neuroimaging complex biomarker discovery [184] it should be possible to provide accurate prognostic models allowing accurate goal setting in neurorehabilitation and stratification in clinical trials [185]. Note that for goal setting in neurorehabilitation, for any patients for whom a given model predicts little potential benefit of treatment, future research will hopefully reveal new, modifiable factors that can be targeted for such patients.

Multivariate machine learning approaches have also been applied to functional MRI data to predict outcome. For example, fMRI data acquired within 2 weeks of stroke in patients with aphasia was used to predict outcome in language [186]. Accuracy in predicting good and bad outcome at 6 months was 76 % and improved to 86 % when age and baseline language impairment were added to the classification model. In the motor domain, fMRI data acquired in the first few days after stroke has been used to try to predict a subsequent change in motor performance [172]. Using a multivariate analysis, a specific pattern of activated voxels was identified as highly predictive of clinical change over the subsequent 3 months, a finding that was independent of initial stroke severity and lesion volume. Anatomical hypotheses could not be tested using this multivariate approach – the study simply indicated that predictive signal was present in a pattern of activation.

#### Predicting treatment effects

Predicting outcome will be useful for clinical and research stratification, but what a clinician would like to know is what are the chances of a patient responding to a specific intervention. Stinear and colleagues [187] set out to determine whether characterising the state of the motor system would help in predicting an individual patient's capacity for further functional improvement at least 6 months post-stroke in a subsequent motor practice programme. A combination of TMS, structural and functional MRI was used to suggest a method for determining who would respond to training. This approach has also been used to predict likely response to robotic training, with both structural [188] and functional imaging [189] data making some contribution.

When it comes to assessing the effects of treatments thought to enhance the potential for experience dependent plasticity, less work has been done. Currently, there is a problematic explanatory gap between molecular (from animal studies) and behavioral (from human studies) accounts of the mechanisms of recovery after stroke. Lack of progression of knowledge from animal models to benefit for human stroke patients has led to the search for ways to study these mechanisms in human subjects. There are exciting advances in how human neuroimaging data can be analyzed that suggest a way forward. Specifically, it is now possible to examine changes in organization of the human brain after stroke at multiple levels of brain architecture, ranging from large-scale networks to alterations in synaptic physiology.

A number of analytical approaches are available to examine connectivity within well-defined macroscopic brain networks. For example, graph theory can be used to determine ‘efficiency’ of information transfer around small world networks such as the brain [190]. This allows inferences to be made about functional connectivity at the level of whole brain, hemisphere or specified network level and can be applied to fMRI, EEG, and MEG data. Dynamic Causal Modeling (DCM) is a method of analyzing data from a dynamic system such as the brain. Bayesian inversion of a specified anatomical model given the empirical data allows the determination of model parameters, which reflect effective connectivity between brain regions (i.e. the task-dependent influence of one brain region on another) [191]. DCM of induced responses acquired with MEG [192] is particularly appealing as it allows partitioning of the effective coupling between regions at the same spectral frequency (linear coupling) and across frequencies (non-linear coupling). Non-linear coupling in particular is important in functional integration [193].

Another recently developed approach to studying brain dynamics after stroke is to use patient-specific structural connectivity data obtained from MRI to set-up an individualized Virtual Brain model of the patient. By optimizing model parameters, such as long-range coupling or local inhibition, in order to match resting state BOLD signals, insight can be gained into how those parameters vary with different types of stroke [194].

At the mesoscopic scale, the spectral characteristics of brain oscillations measured with MEG (or EEG) in the gamma and beta frequency are dependent on the balance of activity between populations of excitatory (glutamatergic) pyramidal cells and inhibitory (GABAergic) interneurons [195] and are candidate biomarkers of the potential for both local- and network-plasticity [196]. More recently, it has become possible to define plausible biophysical DCMs to examine mesoscopic interactions between populations of excitatory and inhibitory cells in specific cortical regions using data from MEG [197]. This approach has been validated using local field potentials in animal preparations where independent pharmacological/microdialysis assays have served to corroborate modelling results [198]. A recent example of how DCM of canonical microcircuits can provide mechanistic inferences is the finding that psilocybin, a 5HT-2A agonist, reduced oscillatory power in posterior association cortex by increasing excitability of deep-layer pyramidal neurons [199].

This range of neuroimaging tools and computational approaches will provide the appropriate intermediate level of description with which to bridge the gap between what we know about recovery after stroke from animal models compared to what we know from studies of behavior in humans. A more detailed knowledge of how these processes are related to impairment and recovery following stroke will provide a mechanistic framework for understanding how to treat patients more effectively. It will open the way for functional brain imaging to become a clinically useful tool in rehabilitation, particularly for our ability to predict outcomes and response to novel plasticity enhancing therapies.

In summary, there is a rich history and promising future to using behavioural status and brain structure and function soon after stroke, to predict long-term motor recovery. Prognostic regression models can inform computational neurorehabilitation models and are a key benchmark against which the predictive power of computational neurorehabilitation will need to be tested.

### Computational neurorehabilitation models

As described above, we use the term “computational neurorehabilitation” to refer to the emergence of theory-driven, mechanistic dynamical models that naturally encode time in differential equations and model recovery of motor behavior using internal states that have a physiologic meaning. These models differ from the prognostic regression models described in the previous section primarily because they incorporate mechanistic dynamical models of plasticity and learning mechanisms underlying recovery. In this section, we critically review a number of recent computational neurorehabilitation models (Table 2).

**Table 2:** Computational neurorehabilitation models discussed in this review

Model A: Han et al. 2008 [112]	
Structure: A bilateral limb-use model using a population vector framework and reinforcement and error-based learning.	
Example Prediction: If spontaneous recovery, motor training, or both, bring function above a certain threshold, then training can be stopped, as the repeated spontaneous arm use provides a form of motor learning that further bootstraps function and spontaneous use (i.e. the “virtuous cycle”)	
Model B: Reinkensmeyer et al. 2012 [136]	
Structure: A wrist strength recovery model using a simplified corticospinal neural network and reinforcement learning via stochastic search	
Example Prediction: Reinforcement learning can explain a broad range of features of stroke recovery, including exponential recovery, residual capacity, and shift of brain activation to secondary motor areas.	
Model C: Casadio and Sanguineti, 2012 [56]	
Structure: An arm impairment reduction model using a linear, discrete-time, shift invariant dynamical system driven by data from robotic therapy	
Example Prediction: A parameter describing retention predicts Fugl-Meyer score 3 months following robotic therapy.	
Model D: Hidaka et al. 2012 [206]	
Structure: First order dynamic model that incorporates a modifiable parameter that controls the effect of arm function on use.	
Example Prediction: Therapy increased the parameter that controls the effect of arm function on use. An increase in this parameter, which can be thought of as the confidence to use the arm for a given level of function, led to an increase in spontaneous use after therapy compared to before therapy.	
Model E: Reinkensmeyer 2003 [207]	
Structure: Adaptive Markov model with Hebbian plasticity that maps relationship between normal and abnormal sensory and motor states, allowing for physical assistance from a rehabilitation trainer	
Example Prediction: Assistance-as-needed can enhance recovery beyond what is possible with unassisted movement practice.	
Model F: Jarrassé et al. 2012 [210]	
Structure: Uses a cost function with error and effort terms, generated by both the therapist (or robot) and human trainee, to characterize a broad range of interactive behaviors of two-agent systems.	
Example prediction: Sensorimotor rehabilitation may be modeled in terms of the cost functions that the trainee and the trainer seek to implement, as well as the algorithms they use to implement those cost functions.	

#### Reaching the threshold for recovery in bilateral hand use

A key aspect of stroke motor recovery is that individuals with a stroke can elect not to use their impaired arm, since they usually have a relatively unaffected arm to perform most needed tasks (i.e. they can compensate). This “learned non-use” may logically be expected to contribute to loss of motor control of the hemiparetic arm (just as an athlete or musician who stops practicing becomes rusty), although to our knowledge there is no research that has yet documented this loss. Han et al. developed one of the first computational neurorehabilitation models to study the interactions between adaptive decision making related to learned non-use and motor relearning after a simulated motor cortex lesion [112] (Fig. 1c). The inputs to the model are targets for bilateral reaching practice and the outputs are the choice of arm to use to reach to the given target, and the kinematic accuracy of the reaching movement. The model incorporates a reward-based learning mechanism for arm selection, and an error-based learning mechanism for refining the neural population code in primary motor cortex that specifies reach direction.

**Fig. 4:**
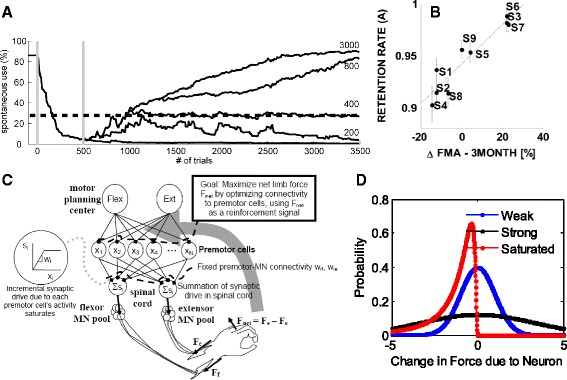
Examples of computational neurorehabilitation approaches and results. **a** A key output of the Han et al. model [112] is the predicted spontaneous use of the impaired hand, shown here as a percent of all movement trials in a bimanual choice task. Each curve represents the evolution of spontaneous use given the number of rehabilitation practice trials, shown as a number on the far right of each curve. Spontaneous use increases only when enough rehabilitation practice trials are performed to reach a threshold. From [112]; used with permission. **b** A key output of the Casadio et al. model [56], which used data from a robotic therapy trial, is that the retention parameter in the model, measured through a trial-to-trial analysis, predicts the change in Fugl-Meyer score at 3 months for these chronic stroke participants. **c** The Reinkensmeyer et al. model [136] assumes that wrist force is produced by the summed effect of corticospinal cells targeting motor neuronal pools. Each cell contributes an incremental force proportional to its firing rate, up to a saturation level. Cell firing rate changes by a random amount from trial to trial; activation patterns that produce more force are remembered for future use, thus implementing a reinforcement learning paradigm. **d** In the Reinkensmeyer et al. model, the probability that a single neuron will contribute to an increase in force on a new trial depends on whether the neuron is strongly or weakly connected to the motor neuronal pool. Strongly connected cells have a greater probability of producing a larger increase. In addition, when cells become saturated, they can only decrease force production. Thus, an increasing number of saturated cells increasingly blocks further optimization, leaving a residual capacity for further increases in force

This model predicts a loss of motor cortex representation without rehabilitation, and a reversal of cortical representational loss with rehabilitation (cf. [93]). Furthermore, the model predicts that if spontaneous recovery, motor training, or both, bring function above a certain threshold, then training can be stopped, as the repeated spontaneous arm use provides a form of motor learning that further bootstraps function and spontaneous use (Fig. 4a), that is, a “virtual cycle” is entered. Below this threshold, motor training is in vain: the model exhibits learned non-use, and compensatory movements performed with the less impaired hand are reinforced; that is, a “viscious cycle” is entered. Evidence for this threshold prediction at the group level was subsequently found in data from the EXCITE clinical trial, a large study of constraint-induced movement therapy after stroke [65].

One clinical impact of the model is that it suggests a novel therapeutic paradigm, the “Train-Wait-Train” paradigm, that not only would test the threshold hypothesis, but also which could potentially be a more cost-effective way to deliver rehabilitation therapy. For example, in computer simulations, a virtual patient was first given a set of 200 trials of therapy, simulated by forced use of the affected arm. Then the patient experienced repeated cycles of 1000 trials in a free arm choice condition (wait), followed by 100 treatment trials (train). The initial training trials were not sufficient to reach threshold, and spontaneous use stayed low. After further training sessions, however, the simulated patient entered the “virtuous circle.” Experiments are currently testing this Train-Wait-Train paradigm.

The model also provides insights into the time constants of stroke motor recovery. The main recovery time constant was on the order of thousands of trials. However, the time constant controlling the change in the decision to use one hand or the other was much smaller, and as a result, the model developed learned non-use soon after stroke. Because how fast learned non-use actually develops after stroke is unknown, hand choice and kinematic data collected soon after stroke would lead to better parameter estimation and hence better predictions from these models.

This model, however, has a number of limitations that need to be addressed to better understand and predict individual recovery as a function of use and motor training. For example, the model is simply bi-stable: either the patient is “above threshold” and fully recovers, or below and does not. In addition, the model ignores interactions between the two cortices. An extension of this model, which includes inter-hemispheric inhibition, has been proposed to account for the beneficial effects of bi-manual training compared to uni-manual training [200]. Further, the model is only concerned with recovery of the control of movement direction, with no attention paid to arm muscle activity, other kinematic features, or functional upper extremity behaviors.

#### Recovering from weakness via reinforcement learning

Motor recovery after stroke is characterized by a seemingly disparate set of behavioral and brain imaging observations, many reviewed above, but could these observations arise from a few fundamental features of human sensorimotor plasticity? Reinkensmeyer et al. approached this question by focusing on the modeling of the recovery of distal upper limb strength, the rationale being that strength strongly predicts upper extremity functional activity [136, 201]. The inputs to the model are attempts to flex the wrist, and the output is the flexion force achieved. For the motor system to recover wrist flexion strength, the model assumes that the motor system must learn appropriate activation of residual corticospinal cells (i.e. those preserved after the stroke – thus, within the terminology adopted in this paper this is again a study of neural compensation not restitution) via repeated movement attempts followed by reward feedback. A simplified corticospinal network model was developed based on several key observations from primate neurophysiological experiments – that corticospinal cell activity sums to create a net excitatory flexor or extensor drive to a joint, that different corticospinal cells have different “gains” for exciting motoneuronal pools, and that the relationship between a corticospinal cell’s activity and its individual contribution to muscle force is linear up to a peak firing rate, then saturates for higher activity levels. Finally, the model assumed that the key underlying mechanism of plasticity driving strength recovery after stroke was a reinforcement learning mechanism, in which the sensorimotor system modifies corticospinal cell activations based on repetitive movement experiences, using stochastic random search, such that limb force output is maximized.

This model predicts a broad range of the features of stroke recovery. It predicts the dose-response curve of rehabilitation, and putative modifiers of this curve, such as that the same dose of exercise has a smaller effect when given to a more severely impaired patient, and that the same dose of exercise given early has a larger differential effect than when given later. This latter finding suggests that increased sub-acute plasticity may arise in part due to normative, compensatory network learning dynamics rather than solely as a function of the stroke-altered tissue microenvironment.

Further, and unlike the Han et al. model reviewed above, the model predicts exponential-like strength recovery curves that never quite reach an asymptote, but instead exhibit a residual capacity for further recovery with further movement practice, as has been observed experimentally [202]. At the level of structural brain imaging observations, the model predicts that patients with a larger residual corticospinal network will recover more, a known stroke phenomenon [203]. It also predicts a key functional imaging observation, which is that movement-related activation in secondary motor areas increases following a stroke that damages output from the primary motor area [204].

Mathematically, two key mechanisms drive the observed network dynamics. First, the saturation in the individual relationships between corticospinal cell firing rates and their contribution to muscle force slows later learning, thus leaving room for residual capacity (Fig. 4d). This is because force results from the summed contributions of many cells. Once the activations of a subset of these cells have evolved in the correct direction to maximize force output, and become saturated, then those neurons can no longer contribute to further increases in force. As saturated neurons randomly vary their output from trial to trial, they exhibit an increased probability of decreasing the force produced on subsequent trials, since they can now only vary their output in the direction counterproductive to further force increase. Second, the activations of more strongly connected cells tend to be modified first, as changes in their activations are more likely to increase force output (Fig. 4c).

A key clinical implication of the model is that, if a single learning mechanism – reinforcement learning via stochastic search – can explain such a broad range of features of stroke recovery, then perhaps this mechanism should be targeted in rehabilitation training to facilitate the search process. Adding variability and chances for exploration to training regimens and providing augmented feedback on the teaching signal are two possible ways to achieve this; a large literature on reinforcement learning in computer science may also be brought to bear on rehabilitation protocol optimization (see above). The model predicts another unexpected way that an improved search might be achieved, an “Inhibit-Train-Release” paradigm, which is to temporarily inhibit cells that are already optimized (perhaps using a localized inhibition scheme, such as infused muscimol or focused transcranial magnetic stimulation, for example), train the remaining cells, and then release the inhibited cells. Stochastic search tends to optimize cells with stronger effects on muscles, and once optimized these cells block further optimization as explained above. The model predicts that temporarily inhibiting optimized cells will allow non-optimized cell activations to become optimized.

As discussed above, this model successfully accounts for a large number of existing neural and behavioral data. However, it has several limitations. It has cartoon-like complexity, modeling only a single flexor and extensor muscle, and simplifying corticospinal activation to static input-output relationships. In addition, it does not model spontaneous recovery or restitution, as we have defined them here. Further, while severe weakness precludes control, mild to moderate weakness is less well correlated with motor control. For example, the ipsilateral limb after stroke shows abnormal motor control but strength is normal [205], so motor control requires more than strength.

#### Robot assistance, retention, and learning predicts recovery

Models such as the Han et al. model or the Reinkensmeyer model above are “qualitative”, i.e., they make general qualitative predictions of recovery. Parameters in these models are “hand-tuned”. As such, they are only loosely based on actual data and cannot be used to predict recovery of individual patients. As reviewed above, during rehabilitative training mediated by a robot it is possible to observe and record the patient’s performance as well as the level of assistance that the patient received. Can the trial-to-trial evolution in movement success during rehabilitation be modeled, and, if so, what are the key learning parameters needed to describe this evolution? Casadio et al. [56] developed the first computational model driven by data from actual robotic training sessions, which describes the trial-by-trial evolution of the recovery process induced by robotic training. This model provides insights into the role of assistive force in the recovery process, and the extent to which learned changes in voluntary control decay over time and transfer to subsequent training sessions.

The model characterizes the recovery process related to robot-assisted training for improving arm extension in chronic stroke survivors as a linear, discrete-time, shift invariant dynamical system. The model posits that motor performance is a function of a voluntary control component, an assistive force component, provided either by a robot or a therapist, and a performance noise term. Thus, the level of physical assistance provided during rehabilitative training is taken as one of the system’s inputs. The voluntary control component is the internal state of the model and its temporal evolution is described as the combination of three additive terms: a ‘memory’ or ‘retention term’, accounting for how much voluntary control depends on previous experience; an ‘assistance’ component expressed as the magnitude of the assistive force; and a learning component that accounts for how much voluntary control on the actual trial is affected by the previous value of the driving signal. The input signals that drive recovery are movement error or a performance measure. A process noise term accounts for the portion of the recovery not due to these three terms.

The most striking result from this model is that the retention parameter predicts the percent change of the Fugl-Meyer score at the 3-month point following the end of the robotic treatment (Fig. 4b). This result, which needs to be confirmed with a larger cohort, is potentially important for the individualization of training. After one or few initial training sessions with the robot, the model can be fitted to the data. Then, by examining the retention parameter, one could potentially determine who will benefit from additional robotic motor training. Note however that this model is based on a set up in which the arm is constrained to move in two dimensions with shoulder and elbow movements. Thus, here again, for example, the issue of restitution versus compensation is difficult to address and the conclusions are limited to simplified movements at only two joints.

#### Understanding interactions between function and use

In the same vein of using computational models for predicting individual recovery, Schweighofer and colleagues developed a first-order dynamical model of stroke recovery with longitudinal data from participants receiving constraint-induced movement therapy in the EXCITE clinical trial [206]. The goal of the model was to better understand the interactions between arm function and use in human post-stroke following therapy. The model shows the existence of time-varying interactions between self-reported amount of daily arm use and recovery of function via a dynamical model of stroke recovery in the 2 years following therapy. The time constants in this model were on the order of several weeks. Of most importance, by comparing the model parameters before and after constraint-induced movement therapy intervention in participants receiving the intervention 1 year after randomization, therapy increased the parameter that controls the effect of arm function on use. An increase in this parameter, which can be thought of as the confidence to use the arm for a given level of function, led to an increase in self-reported spontaneous use after therapy compared to before therapy. Thus, here, the parameters of the model can be viewed as “states” that have physiological meaning, which may expedite the testing of new experimental hypotheses.

However, as in the previous model of Casadio et al., this is a highly simplified model of recovery. It contains only a few parameters and two time constants, one for functional recovery and the other for hand use. The reason for this simplicity is the very sparse data set on which the model was built. Increasing the complexity of the model, and thus the number of parameters was not possible because this would have led to overfitting, i.e., the model would fit the data very well but would not generalize.

#### Modeling the effect of assistance-as-needed

Rehabilitation therapy often involves interactions between patient and a trainer, be it a rehabilitation therapist, a robotic device, or a computer game. Modeling the patient-trainer interaction could potentially provide insight into movement recovery.

Reinkensmeyer used an adaptive Markov model to examine the role of external mechanical assistance from a robotic device or therapist in promoting movement recovery [207]. Following research in gait training after spinal cord injury, the model assumes that motor control is characterized by repeated transitions between sensory and motor states; for example, sensing of full hip extension at the end of stance triggers leg flexion. It further grossly characterizes these states as abnormal and normal sensory states (e.g. appropriate or inappropriate hip extension), and abnormal and normal motor states (effective or ineffective leg flexion). The model uses a Hebbian-inspired model of plasticity in which the transitions between specific sensory and specific motor states become more reliable with repetitive activation of that transition. The action of a skilled external trainer (robot or human) who is assisting in movement is modeled as a mediated increase in the probability of transferring to a state of normal motor output (e.g. the trainer helps generate hip extension sensory inputs to enable effective leg flexion). Assistance-as-needed is simulated by mediating this transfer only when the patient is in an abnormal motor state, while assistance-always is simulated by mediating this increase on every movement repetition.

The model predicts that assistance-as-needed can enhance recovery beyond what is possible with unassisted practice, that assistance-always is not as effective as assistance-as-needed, that the trainer’s skill in assisting toward normal motor output matters in reinforcing normal state transitions, and that assistance is not useful when sensory input is less directly coupled to motor output. While these predictions may sound somewhat intuitive, this is perhaps because they mirror opinions that are often expressed in the current clinical milieu. The model in this case served to verify that these opinions could be mathematically supported relying on a simple but plausible plasticity rule.

#### Patient-trainer dynamics as an optimization

Another approach toward modeling patient-trainer dynamics is based on what was first developed as a model of human movement adaptation. As briefly described above, the motor system uses a sunken-v muscle adaption rule to alter force, impedance, and trajectory, explaining a wide variety of experimental findings of reaching in dynamic environments [119, 208]. This update rule can be viewed as the motor system implementing a greedy minimization of a cost function of error and effort [118]. If the patient’s motor system is minimizing a cost function from movement to movement, then, what cost function might the trainer minimize to assist in learning? A gradient descent of a similar cost function of error and effort was shown to provide an efficient assistive controller for rehabilitation [209]. Jarrassé et al. expanded and formalized this approach, providing a framework for the description and implementation of a broad range of interactive behaviors of two agents (such as a patient and a robotic therapist) performing a joint motor task, including rehabilitation [210]. For example, one may assume that a robotic trainer just moves a patients’ limb while this patient is passive. To model this situation, the trainer would take all of the effort off of the patient by minimizing its cost function:

3 $$ {\mathrm{V}}_{\mathrm{t}}={\upalpha \mathrm{e}}^2+{\upbeta \mathrm{u}}^2,\upalpha, \upbeta >0, $$

where e and u correspond to the patient’s error and effort state as observed by the trainer. However, individuals with stroke improve their motor function more when actively attempting to move, rather than relying on the robot to move their arm [211]. To avoid the patient becoming passive, the trainer can modify its cost function to

4 $$ {\mathrm{V}}_{\mathrm{t}}={\upalpha \mathrm{e}}^2+{\upbeta}_{\mathrm{t}}{{\mathrm{u}}_{\mathrm{t}}}^2,\upalpha, {\upbeta}_{\mathrm{t}}>0 $$

i.e. minimize its own effort u_t_^2^. If one assumes that the patient behaves according to the sunken-v adaptation rule, if the trainer is slightly more lazy (i.e. β_t_ > β), it will help the patient fulfill the task (as the trainer also has to minimize e^2^) only if the patient is not able to do so by itself, and disappear if the patient can do it. Thus, this method and the cost function of Eq. (4) can be used to design assist-as-needed control of rehabilitation robots. In terms of computational neurorehabilitation, this work suggests that sensorimotor rehabilitation may be able to be modeled in terms of the cost functions that the trainee and the trainer seek to implement, as well as the algorithms they use to implement those cost functions.

## Conclusions

We contend that the models reviewed in the previous section are evidence that a fundamental understanding of neurologic recovery will be facilitated by modeling motor learning and plasticity itself in a context specific to rehabilitation. Even the initial, simplified models presented are generating novel ideas concerning the mechanisms of recovery in rehabilitation, and thus are suggesting important directions for future experimental research.

### Novel ideas from initial models and their limitations

For example, the Han et al. model discussed above suggests the existence of a threshold of movement ability that allows patients to enter a “virtuous” cycle of activity-dependent plasticity; this premise is now being tested in a clinical trial. The Reinkensmeyer et al. model suggests that a large number of existing behavioral and neural data can be accounted for if the motor system uses a reinforcement-learning paradigm – stochastic search – to optimize neural activations; this suggests increasing research into techniques to enhance reinforcement learning in order to enhance rehabilitation therapy, a proposition that is only beginning to be explored. The Casadio et al. model showed that a short-term measure of retention in robotic training can accurately predict how much long-term recovery is possible with such training during chronic stroke, suggesting a novel assay for patient selection for robotic therapy. Other interesting ideas generated by the initial models reviewed above are that a key way that rehabilitation therapy may function is by modulating a parameter that controls the effect of arm function on use, and that trainer-trainee interactions can be characterized using parsimonious cost functions.

Even given their utility in generating such novel ideas concerning rehabilitation, these initial models clearly have shortcomings. They greatly simplify human movement and the rehabilitation process. They ignore the fact that every day activity relies on bimanual motor function. They selectively model only one or two learning and plasticity mechanisms. They focus on motor learning rather than plasticity mechanisms associated with restitution, as yet neglecting the rich literature on spontaneous biological recovery, which may ultimately offer greater potential for recovery. They have not yet been used to replicate, let alone extend, the findings achieved with prognostic regression models. None have yet used a cross-validation procedure in order to see how much a model based on the data from a pool of patients can explain the behavior of patients outside of that pool. In addition, sensorimotor control is just one of many domains addressed by neurorehabilitation.

### The forthcoming data revolution

Another key argument that this review has attempted to make is that the time is propitious for continued development of computational neurorehabilitation models. As we surveyed above, the mathematical tools for developing such models are already at least partly available because of the past several decades of work in the field of computational neuroscience and machine learning. Further, as we also reviewed above, large-scale longitudinal data for each patient and for a large number of patients are now possible to obtain with robotic devices and wearable sensors. This will allow the development of more elaborate, physics-based models that predict recovery at a fine temporal and spatial resolution. Note that conventional motor learning experiments have generally failed to even come close to the number of training trials that gives rise to the high level expertise in sports and work-related skills. With instrumented rehabilitation and wearable sensors, this situation is now set to change, at least in the context of motor learning during rehabilitation. A key challenge for the future is how to utilize these longitudinal data that are collected on different samples, with different methods of motion capture, and different quantifications of movement into a coherent package such that they can inform models of movement control over the course of recovery after stroke.

The Casadio et al. model described above initiates the data-driven approach, as it is the first to use trial-to-trial data from actual robotic training to predict changes in Fugl-Meyer scores, suggesting that retention-related parameters correlate with impairment recovery. Following this approach, we predict that soon we will see the emergence of large scale mechanistic models of motor recovery that are driven by the actual movement content of rehabilitation therapy as well as records of daily activity. Ideally, one would use the same data set from hundreds or even thousands of well-characterized patients, so that computational model output is realistic and appropriate.

In addition, the models will increasingly be informed by automatic analysis of MRI scans routinely obtained after stroke, since, as we reviewed above, brain anatomical information is needed because identical behavior can arise through largely different neural processes. A range of novel neuroimaging tools and computational methods, including analysis of grey and white matter structures and structural and functional connectivity [212], will provide an intermediate level of description with which to bridge the gap between what we know about recovery after stroke from animal models compared to what we know from studies of behavior in humans.

### Complexification and utility

Clearly, initial computational neurorehabilitation models are vast simplifications of a very complex process. While simplification and abstraction are often virtues in modeling, with richer data sets, it will be possible to increase the complexity of computational neurorehabilitation models with less risk of overfitting. Models containing a multiple joint system, such as the whole arm or both arms, will be important for understanding compensation. Upper extremity motor control during real-world, free-living activity involves the movement of all the segments in order to position and orient the limb and to interact with objects [213, 214]. If a computational model utilizes only a few of the segments, then the model output will provide only a limited view of the actual solution, essentially ignoring the degrees of freedom problem underscored by Bernstein. Models that can help understand or predict control processes during naturalistic actions will be of high value for the field of neurorehabilitation.

Further, it is currently difficult to experimentally study the interacting effects between different forms of learning, such as supervised, unsupervised, and reinforcement learning, including the role of the timing of rehabilitation on these processes [81, 215]. There appear to be methods also to induce beneficial plasticity beyond task repetition, such as the possible enhancing of effects of non-invasive brain stimulation on motor learning [216, 217]. Computational neurorehabilitation models can incorporate multiple levels of plasticity and learning, as well as plasticity-enhancing effects of techniques such as electrical stimulation, and even psychological effects important to rehabilitation, helping understand these interactions in computer simulations to guide future experimental work. This is important, as one theoretically could vet hypotheses in an efficient and cost-effective manner, rather than relying solely on randomized, controlled trials, which are costly and time consuming.

A key question, of course, is whether the incorporation of plasticity and learning mechanisms, along with internal physiological states, into models will improve upon the predictive capability now possible with prognostic regression models. We contend they have a good chance to, because they are more likely to isolate the key predictive variables of interest, since these variables likely relate to physiologic function, and computational neurorehabilitation models seek to make just such variables explicit. Such variables likely vary from patient-to-patient as well, suggesting that their isolation will improve individualized predictions.

If so, computational models of neurorehabilitation should ultimately improve rehabilitation for individuals with neurologic injuries. We expect that computational models of recovery, based on early clinical data, kinematic performance, and routine scans, will provide the basis for future clinical software that suggests timing, dosage, and content of therapy. Such an approach will transform neurorehabilitation by guiding clinicians, patients, and health providers in the optimization of treatments.
